# Type studies of *Rossbeeverabispora*, and a new species of *Rossbeevera* from south China

**DOI:** 10.3897/mycokeys.51.32775

**Published:** 2019-03-11

**Authors:** Md. Iqbal Hosen, Xiang-Jing Zhong, Genevieve Gates, Takamichi Orihara, Tai-Hui Li

**Affiliations:** 1 State Key Laboratory of Applied Microbiology Southern China, Guangdong Provincial Key Laboratory of Microbial Culture Collection and Application, Guangdong Institute of Microbiology, Guangzhou 510070, Guangdong, China Guangdong Institute of Microbiology Guangzhou China; 2 Administration of Guangdong Xiangtoushan National Nature Reserve, Huizhou 516001, Guangdong, China Administration of Guangdong Xiangtoushan National Nature Reserve Huizhou China; 3 Tasmanian Institute of Agriculture, Private Bag 98, Hobart, Tasmania 7001, Australia Tasmanian Institute of Agriculture Hobart Australia; 4 Kanagawa Prefectural Museum of Natural History, 499 Iryuda, Odawarashi, Kanagawa 250-0031, Japan Kanagawa Prefectural Museum of Natural History Odawarashi Japan

**Keywords:** Boletaceae, East Asia, multi-locus phylogeny, new taxon, taxonomy

## Abstract

The type of *Rossbeeverabispora* and additional collections from the type location and adjacent areas were studied. Molecular data for *R.bispora* derived from the new collections are provided. In addition, *R.griseobrunnea*, a new species of *Rossbeevera*, is described from Xiangtoushan National Nature Reserve, Guangdong Province of China. The new species is characterized by its globose to subglobose sequestrate basidiomata with grayish white to grayish brown pileus, pale bluish discoloration in some parts of the hymenophore when injured becoming rusty brown to dark brown after being exposed to the air, fusoid (star-shaped in cross section) basidiospores 17–20 × 9–12 μm, and subcutis elements in the pileus. Based on multi-locus (ITS+nrLSU+*tef1-α*+*rpb2*) molecular phylogenetic analyses, both species appear as sister to *R.paracyanea*. We present color photos, macro- and micro-description, SEM basidiospores, molecular affinities of the species and compare them with morphologically similar taxa within the genus. A key to the species known from northern and southern hemispheres is provided.

## Introduction

*Rossbeevera* T. Lebel & Orihara, a sequestrate ectomycorrhizal genus of Boletaceae, was erected in 2012 to accommodate *Chamonixiapachydermis* Zeller & C.W. Dodge as the type of the genus (Lebel et al. 2012). The genus represents a distinct monophyletic group in the subfamily Leccinoideae, and is strongly supported as a sister to another sequestrate genus *Turmalinea* Orihara & N. Maek. (Orihara et al. 2016). Species of *Rossbeevera* are easy to recognize in the field as they have rubbery sequestrate basidiomata, with or without bluish discoloration of either the pileus or the hymenophore and usually a thin pileipellis. Microscopically, they have a hymenium that is developed when the basidiomata is immature and collapses at maturity, and ornamented basidiospores with 3–5 longitudinal ridges (Lebel et al. 2012, Orihara et al. 2016).

Presently *Rossbeevera* includes 10 species (Lebel et al. 2012, Orihara et al. 2012, 2016), only known from Asia (China, Japan and Singapore/Malaysia) and Australasia (Australia and New Zealand). Prior to this study, two of them (*R.bispora* (B.C. Zhang & Y.N. Yu) T. Lebel & Orihara and *R.yunnanensis* Orihara & M.E. Sm.) were known from China (Zhang and Yu 1989, Orihara et al. 2012). In 2018, Orihara reported *R.yunnanensis* from Japan. Of the remaining eight species: four (*R.cryptocyanea* Orihara, *R.eucyanea* Orihara, *R.griseovelutina* Orihara and *R.paracyanea* Orihara) are known from Japan (Lebel et al. 2012; Orihara et al. 2016), two (*R.vittatispora* (G.W. Beaton, Pegler & T.W.K. Young) T. Lebel and *R.westraliensis* T. Lebel, Orihara & N. Maek) from Australia (Lebel et al. 2012), one (*R.pachydermis* (Zeller & C.W. Dodge) T. Lebel) (orthographic variant *R.pachyderma*) from New Zealand (Lebel et al. 2012) and one (*R.mucosa* (Petri) T. Lebel, Orihara & N. Maek.) from Singapore/Malaysia (Lebel et al. 2012, Orihara et al. 2016).

In this study, several collections of *Rossbeevera* resembling *R.bispora* have repeatedly been found in south China (Guangdong Province: Dinghushan Nature Reserve, a type locality of *R.bispora*; Baiyunshan Mountain, Tianluhu Park and Xiangtoushan National Nature Reserve). Among the collections examined in this study two of them appeared to be clearly different from *R.bispora* (although all collections were originally treated as *R.bispora*). Therefore, we studied the type material of *R.bispora* for comparison with the recent collections. A combination of morphological data and multi-locus phylogenetic analyses including sequences of the nuclear ribosomal internal transcribed spacer (ITS) region, nuclear ribosomal large subunit (nrLSU), translation elongation factor 1-α (*tef1-α*), and the second largest subunit of RNA polymerase II (*rpb2*) were used for the delimitation of a new species within the genus *Rossbeevera*.

## Materials and methods

### Sampling and morphological studies

The specimens were collected from south China (Guangdong Province: Dinghushan Nature Reserve, Tianluhu Park, Baiyunshan Mountain and Xiangtoushan National Nature Reserve). After being examined and described the dried specimens were deposited in the Fungal Herbarium of the Guangdong Institute of Microbiology, Guangzhou, China (GDGM).

Macromorphological descriptions were based on field notes and photographs. Micromorphological observations were made from small pieces of dried specimens mounted in H_2_O, 5% aqueous KOH (w/v), Congo Red and Melzer’s solution. In the description of the basidiospore measurements, the notation [n/m/p] is used, which means *n* basidiospores from *m* basidiomata of *p* collections. Dimensions for basidiospores are given as (a–)b–c(–d), in which ‘b–c’ contains a minimum of 90% of the measured values and extreme values ‘a’ and ‘d’ are given in parentheses, whenever necessary. Q denotes the length/width ratio of a measured basidiospore, Q_m_ denotes the average of *n* basidiospores, and SD is their standard deviation. Results are presented as Q_m_ ± SD. For describing the species, we used the taxonomic terminology pileus for ‘peridium’, hymenophore for ‘gleba’ and stipe for ‘columella’.

### Molecular studies

Protocols for genomic DNA extraction, PCR amplification, and sequencing followed Hosen et al. (2013) and references therein. The ITS1-F/ITS4 (White et al. 1990), LROR/LR5 (Vilgalys and Hester 1990), ef1-983F/ef1-1567R (Rehner and Buckley 2005) and rpb2-B-F/rpb2-B-R (Wu et al. 2014) primer pairs were used for the amplification of ITS, nrLSU, *tef1-α* and *rpb2* regions.

Currently molecular data are available for eight of the 10 reported species. The final dataset consisted of 10 species of *Rossbeevera* including *R.bispora* and a new species (see taxonomy). Representative sequences (ITS, nrLSU, *tef1-α* and *rpb2*) of *Rossbeevera* and its allied genera from the subfamily Leccinoideae were retrieved from GenBank. Individual gene fragments were aligned in MAFFT v.6.8 (Katoh et al. 2005), and manually edited in BioEdit v.7.0.9 (Hall 1999) using default settings. Prior to concatenating the multi-locus (ITS+nrLSU+*tef1-α*+*rpb2*) dataset, an individual aligned dataset was analyzed separately to detect the topologies (BS ≥70%). There was no significant incongruence detected while reconstructing ITS, nrLSU or ITS+nrLSU/ITS+nrLSU+*tef1-α* except for the individual dataset of *tef1-α* and *rpb2* (because of the lack of representative *tef1-α* and *rpb2* sequences for *Rossbeevera* species) phylogenetic trees. A multi-locus dataset was built using Phyutility (Smith and Dunn 2008) for further phylogenetic analyses, and the resulting dataset was deposited in TreeBASE (S23404). Maximum Likelihood (ML) was used to analyze the multi-locus dataset. ML was performed in RAxML v.7.2.6 (Stamatakis 2006) with default settings. Statistical support values were obtained using nonparametric bootstrapping (BS) with 1000 replicates.

## Results

### Molecular phylogenetic results

**Figure 1.**
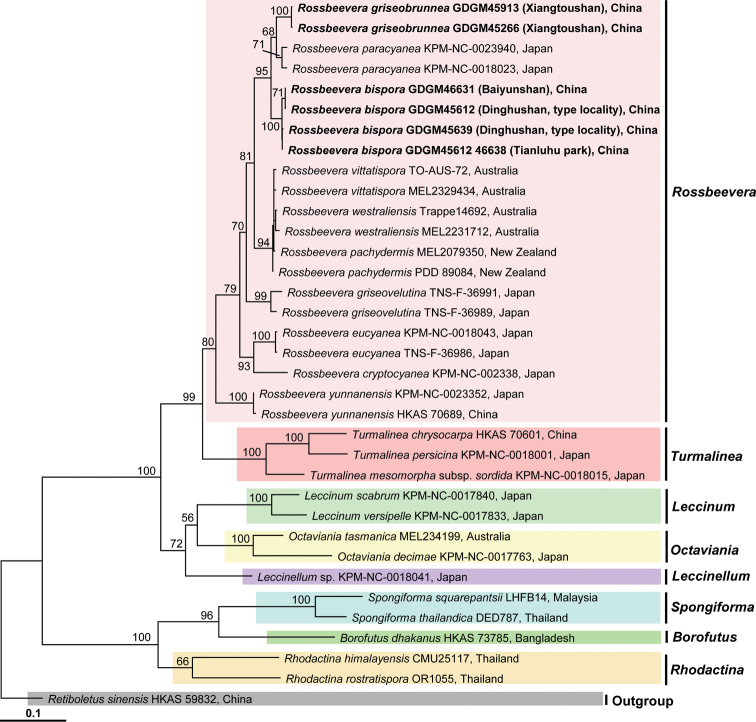
Phylogenetic relationships of *Rossbeevera* and its allied genera inferred from multi-locus (ITS+nrLSU+*tef1-α*+*rpb2*) analyses. *Rossbeeverabispora* and *R.griseobrunnea* are highlighted in bold on the tree. RAxML bootstrap (BS) support values (MLBS>50%) are indicated on the branches at nodes. Voucher number/collection number are provided after each species followed by country name.

In this study, 15 new sequences were generated from the Chinese collections of *Rossbeevera* and deposited in GenBank (Table 1). The combined aligned dataset included 35 specimens from 24 species in the Boletaceae (10 of *Rossbeevera*, three of *Turmalinea*, two each of *Leccinum* Gray, *Octaviania* Vittad., *Rhodactina* Pegler & T.W.K. Young and *Spongiforma* Desjardin, Manfr. Binder, Roekring & Flegel, and one each of *Borofutus* Hosen & Zhu L. Yang, *Leccinellum* Bresinsky & Manfr. Binder and *Retiboletus* Manfr. Binder & Bresinsky). The combined alignment contained 3928 nucleotide sites (gaps included) for each sample, of which 1118 were ITS, 916 were nrLSU, 1128 were *tef1-α* and 766 were *rpb2*. *Rossbeeverabispora* is nested in a clade containing *R.paracyanea* and *R.griseobrunnea* with strong support (95% MLBS, Fig. 1). Two collections of *R.griseobrunnea* (GDGM 45266 and GDGM 45913) formed a monophyletic clade and sister to the Japanese *R.paracyanea* with moderate support (68% MLBS, Fig. 1). Interestingly, these three East Asian species formed a sister clade with the Australasian *Rossbeevera* species including *R.westraliensis*, *R.vittatispora* and *R.pachydermis* with moderate strong support (81% MLBS, Fig. 1). The summarized result of the phylogenetic analysis is presented in Fig. 1.

**Table 1.** List of fungal taxa of Boletaceae with voucher number, country of origin and GenBank accession numbers used in the molecular phylogeny.

**Name of the species**	**Voucher/collection no.**	**Country**	**GenBank accession number**			
			**ITS**	**nrLSU**	***tef1*-α**	***rpb2***
* Borofutus dhakanus *	HKAS 73785*	Bangladesh	JQ928605	JQ928615	JQ928577	JQ928596
*Leccinellum* sp.	KPM-NC-0018041	Japan	–	KC552053	KC552094	–
* Leccinum scabrum *	KPM-NC-0017840	Japan	KC552012	JN378515	JN378455	–
* Leccinum versipelle *	KPM-NC-0017833	Japan	–	JN378514	JN378454	–
* Octaviania decimae *	KPM-NC-0017763	Japan	JN257991	JN378465	JN378409	–
* Octaviania tasmanica *	MEL2341996	Australia	KC552004	JN378495	JN378436	–
* Retiboletus sinensis *	HKAS 59832	China	–	KT990633	KT990827	KT990464
* Rhodactina himalayensis *	CMU25117	Thailand	–	–	MG212603	–
* Rhodactina rostratispora *	OR1055	Thailand	–	–	MG212604	MG212644
* Rossbeevera westraliensis *	Trappe14692	Australia	HQ647131	HQ647153	–	–
* Rossbeevera bispora *	GDGM 45612	China	**MK035705**	**MK036346**	–	**MK350308**
* Rossbeevera bispora *	GDGM 45639	China	–	**MK036347**	–	**MK350309**
* Rossbeevera bispora *	GDGM 46631	China	**MK035705**	**MK036348**	–	–
* Rossbeevera bispora *	GDGM 46638	China	–	**MK036349**	–	–
* Rossbeevera cryptocyanea *	KPM-NC0023387	Japan	KP222893	KP222899	KP222913	–
* Rossbeevera eucyanea *	KPM-NC-0018043	Japan	KC551983	KC552029	KC552071	–
* Rossbeevera eucyanea *	TNS-F-36986*	Japan	HQ693875	HQ693880	KC552068	–
* Rossbeevera griseobrunnea *	GDGM 45266	China	**MH532533**	**MH537792**	–	**MK350310**
* Rossbeevera griseobrunnea *	GDGM 45913*	China	**MH532534**	**MH537793**	**MK350307**	**MK350311**
* Rossbeevera griseovelutina *	TNS-F-36989*	Japan	HQ693876	KC552031	KC552076	–
* Rossbeevera griseovelutina *	TNS-F-36991	Japan	KC551985	KC552032	KC552077	–
* Rossbeevera pachydermis *	MEL2079350	New Zealand	HQ647138	HQ647157	–	–
* Rossbeevera pachydermis *	PDD:89084	New Zealand	GU222301	–	–	–
* Rossbeevera paracyanea *	KPM-NC-0018023	Japan	KC551988	KC552035	–	–
* Rossbeevera paracyanea *	KPM-NC0023940	Japan	KP222894	–	–	–
* Rossbeevera vittatispora *	MEL2329434	Australia	KJ001084	KJ001097	KJ001075	–
* Rossbeevera vittatispora *	TO-AUS-72	Australia	KC551977	KC552025	KC552065	–
* Rossbeevera westraliensis *	MEL2231712	Australia	HQ647140	HQ647162	–	–
* Rossbeevera yunnanensis *	HKAS 70689*	China	–	JN979437	–	–
* Rossbeevera yunnanensis *	KPM-NC 23352	Japan	MF357925	MF354015	–	–
* Spongiforma squarepantsii *	LHFB14	Malaysia	HQ724511	HQ724509	–	–
* Spongiforma thailandica *	DED7873*	Thailand	EU685113	EU685108	KF030436	KF030387
* Turmalinea chrysocarpa *	HKAS 70601*	China	–	KF112448	–	KF112729
Turmalinea mesomorpha subsp. sordida	KPM-NC-0018015*	Japan	KC552001	KC552049	KC552092	–
* Turmalinea persicina *	KPM-NC-0018001*	Japan	KC551991	KC552038	KC552082	–

Highlighted in bold are newly generated sequences in this study. *holotype. En dash (–) indicates information is not available.

### Taxonomy

#### Rossbeevera bispora

Taxon classificationFungiBoletalesBoletaceae

(B.C. Zhang & Y.N. Yu) T. Lebel & Orihara, Fungal Diversity 52(1): 58 (2012)

Figs 2 , 3 , 6a, b

≡ Chamonixiabispora B.C. Zhang & Y.N. Yu, Mycotaxon 35(2): 278 (1989).

##### Description.

*Basidiomata* hypogeous, 25–45 mm broad, 20–30 mm high, small, globose to subglobose, napiform, sometimes deformed or reniform, fleshy when fresh, firm when dry. *Pileus* thin, surface glabrous to slightly velvety in some cases, shiny, sometimes alveolate or cracking with age, dull white, grayish white to grayish brown, whitish at the lower portion, turning blue to deep blue when touched or injured or when mature, occasionally basal part covered with yellowish white mycelia. *Hymenophore* off-white, white to dull white when young, blue to dark blue immediately when cut or injured, fleshy, soft and smooth, composed of minute, irregular locules, becoming partially collapsed with many small empty chambers when dried. *Stipe* absent. *Sterile base* present but reduced, white, dull white to grayish white, somewhat dendroid or as a small basal pad or rhizomorph. *Odor and taste* not recorded.

*Basidiospores* [80/4/4] 16–21 × 9–11.5 μm [mean 18.55 × 10.58 μm, Q = 1.63–1.83(–1.90), Q_m_ = 1.75 ± 0.11], fusoid, ornamented with 4-longitudinal ridges (star-shaped in cross section), inamyloid, brown to dark brown in KOH and H_2_O, thick-walled up to 2 μm thick, hilar appendages 1–3 μm long. *Basidia* 12–25 × 5–8 μm, narrowly clavate to cylindro- clavate, hyaline to pale yellow, usually 2-spored, occasionally 1-spored. Hymenial cystidia absent. *Hymenium* developed when immature but collapsed at maturity, hyaline to pale yellowish; subhymenium not developed. *Hymenophoral trama* 60–160 μm wide, subgelatinous, composed of hyaline, cylindrical, loosely interwoven to parallel, frequently branched, thin-walled, cylindrical hyphae 2–5 μm wide. *Pileipellis* a repent cutis, terminal cells short, clavate to cylindro-clavate, yellowish brown to brownish pigmented, smooth, thin-walled. *Clamp connections* absent in all tissues.

##### Habit and habitat.

Solitary or in small groups beneath or on the ground, hypogeous or partially epigeous in a subtropical evergreen broad-leaved forest, putatively associated with *Castanopsisfissa* Rehder & E.H.Wilson, *C.chinensis* (Spreng.) Hance, *C.fabri* Hance and *Schimasuperba* Gardner & Champ.

##### Known distribution.

Currently known from south China (Guangdong Province: (Baiyunshan Mountain, Dinghushan Nature Reserve and Tianluhu Park).

##### Specimens examined.

CHINA. Guangdong Province, Zhaoqing City, Dinghushan Nature Reserve, 13 October 1982, You-Zao Wang, Wan-Ling Zhen, Jinag-Qing Li (GDGM 5688, holotype); 7 March 2013, Karl (GDGM 45612 and GDGM 45639); Baiyun Mountain, 7 April 2017, Yong He (GDGM 46631); Tianluhu Park, 21 March 2018, Tai-Hui Li, Chenghua Zhang, Xishen Liang (GDGM 46638).

##### Comments.

**Figure 2.**
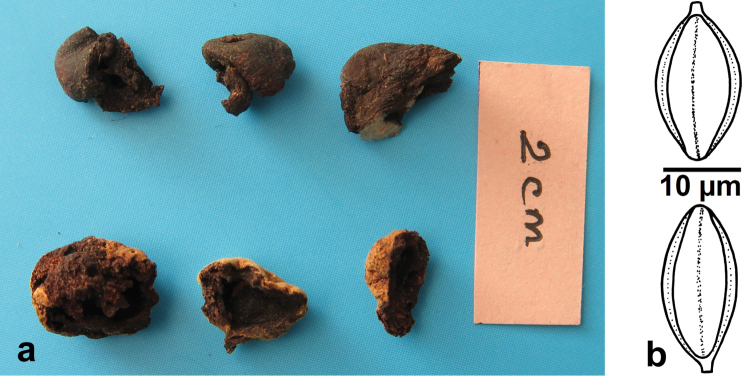
Type specimen of *Rossbeeverabispora* (as *Chamonixiabispora*, GDGM 5688, holotype) **a** dried basidiomata of *Rossbeeverabispora***b** basidiospores.

Zhang and Yu (1989) described *R.bispora* as *Chamonixiabispora* B.C. Zhang & Y.N. Yu from south China (Dinghushan Nature Reserve, Guangdong Province) based on a single collection (Fig. 2). Lebel et al. (2012) transferred this species to *Rossbeevera* as it fits within the generic concept of *Rossbeevera*. This species is characterized by its whitish to grayish brown pileus turning bluish when injured, 2-spored basidia, and is associated with broad-leaved trees in south China.

**Figure 3.**
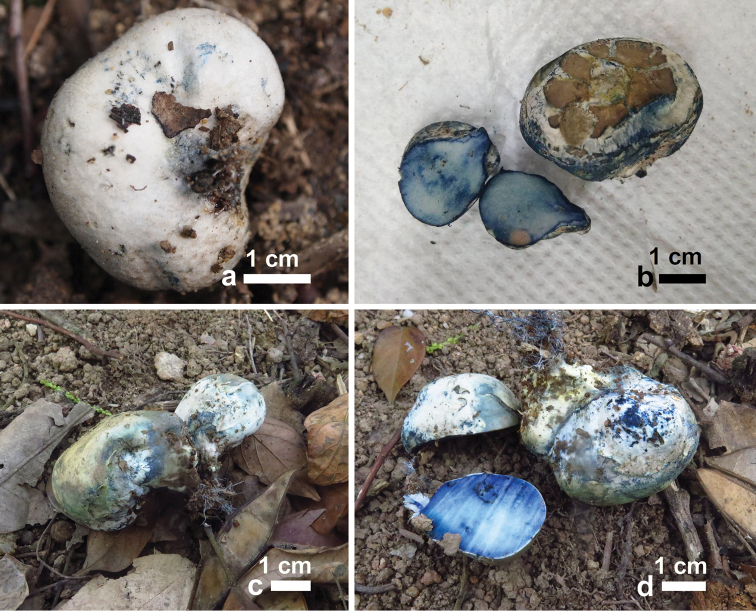
Basidiomata of *Rossbeeverabispora* (new collections). **a** Dull white to grayish brown pileus with blue tinges in some portion (GDGM 45612) **b** Bluing hymenophore (after injured) and pileus surface (GDGM 46631) **c** Bluing pileus with reduced stipe (GDGM 46638) **d** Bluing hymenophore (after injured) and pileus surface (GDGM 46638).

#### Rossbeevera griseobrunnea

Taxon classificationFungiBoletalesBoletaceae

Iqbal Hosen & T.H.Li sp. nov.

MB826880

Figs 4 , 5 , 6c, d

##### Diagnosis.

Basidiomata hypogeous, small; pileus grayish white to grayish brown, surface bluing slightly when injured; hymenophore dull white to very pale blue in some portion when injured, finally rusty brown to dark brown at maturity; stipe absent; basidiospores 17–20 × (8–)9–12 μm, fusoid, ornamented with 4-longitudinal ridges (star-shaped in cross section), brown to dark brown; pileipellis a subcutis, with terminal elements short cylindro-clavate.

##### Typification.

CHINA. Guangdong Province, Boluo County, Xiangtoushan National Nature Reserve, 19 November 2015, Tai-Hui Li, Ting Li, Hao Huang & Jun-Ping Zhou (GDGM 45913, holotype).

##### Etymology.

The epithet name ‘*griseobrunnea*’ (Lat.) refers to the grayish brown pileus.

##### Description.

*Basidiomata* hypogeous, 15–35 mm broad, 12–25 mm high, small, globose to subglobose, napiform, sometimes deformed or reniform, fleshy when fresh, firm when dry. *Pileus* very thin, surface glabrous to slightly velvety, shiny, grayish white to grayish brown, whitish at the lower portion, turning to pale blue when touched or injured. *Hymenophore* off-white, white to dull white when young, becoming pale blue to bluish in some parts/patches then rusty brown to dark brown when exposed to air for 3–5 minutes, often greenish brown around insect damage, firm, composed of minute, irregular locules, becoming partially collapsed with many small empty chambers when dried. *Stipe* absent. *Stipe* base present but reduced, white, dull white to grayish white, somewhat dendroid or as a small basal pad or rhizomorph. *Odor and taste* not recorded.

*Basidiospores* [50/2/2] 17–20(−21) × (8–)9–12(–13) μm [mean = 18.5 × 10.5 μm, Q = (1.52–)1.63–1.91(–2.1), Q_m_ = 1.81 ± 0.18] fusoid, ornamented with 4-longitudinal ridges (star-shaped in cross section) (up to 2.5 μm high), inamyloid, non-dextrinoid, brown to dark brown in KOH and H_2_O, thick-walled up to 2 μm thick, hilar appendages 1.5–3 μm long. *Basidia* 15–27 × 5–9 μm, narrowly clavate to cylindro- clavate, hyaline to pale yellow, usually 2-spored, occasionally 1-spored; basidioles 18–25 × 8–10 μm, clavate to short clavate. *Hymenial* cystidia absent. *Hymenium* developed when immature but collapsed at maturity, hyaline to pale yellowish; subhymenium not developed. *Hymenophoral trama* 60–130 μm wide, subgelatinous, composed of hyaline, cylindrical, loosely interwoven to parallel, frequently branched, thin-walled, cylindrical hyphae 2–5 μm wide. *Pileipellis* a subcutis with terminal elements 15–20 × 7–9 μm, short clavate to cylindro-clavate, yellowish brown to brownish pigmented, smooth, thin-walled. *Clamp connections* absent in all tissues.

##### Additional specimen examined.

CHINA, Guangdong Province, Boluo County, Xiangtoushan National Nature Reserve, 19 November 2015, Tai-Hui Li, Ting Li, Hao Huang & Jun-Ping Zhou (GDGM 45266).

##### Habit and habitat.

Solitary or in small groups beneath or on the ground, hypogeous or partially epigeous in a subtropical evergreen broad-leaved forest, putatively associated with *Castanopsisfissa*, *C.chinensis*, *C.fabri* and *Schimasuperba*.

##### Known distribution.

Currently known only from south China (Guangdong Province, Xiangtoushan National Nature Reserve).

##### Comments.

The voucher specimen (GDGM 45266) was cited as *Rossbeevera* sp. by Hosen et al. (2018), while studying boletes from the Xiangtoushan National Nature Reserve, Guangdong Province, China. After morphological and molecular comparisons with other known species of *Rossbeevera* the voucher specimen (GDGM 45266) is described here as *R.griseobrunnea*. It is characterized by its dull white, grayish white to grayish brown basidiomata, with hymenophore that discolors very slowly in some portions (pale blue in some patches becoming rusty brown to dark brown) after being cut or injured, fusoid ornamented basidiospores with mostly 4-longitudinal ridges and subcutis elements (short clavate terminal cells) in the pileus.

**Figure 4.**
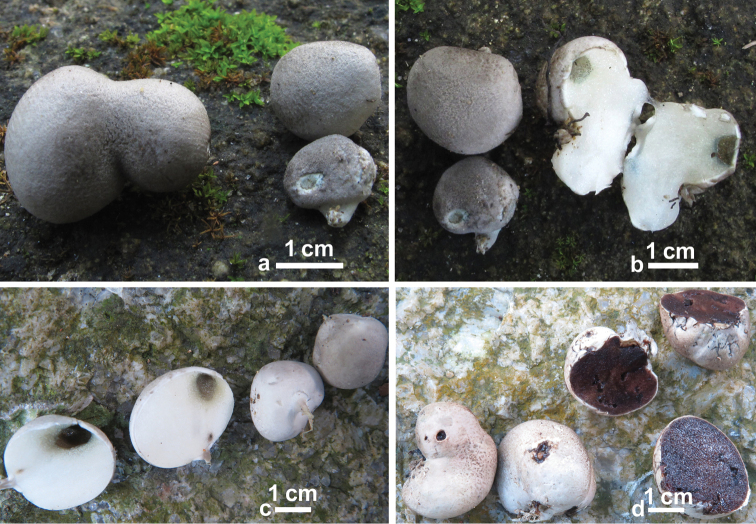
Basidiomata of *Rossbeeveragriseobrunnea*. **a** Unchanged pileus surface with reduced stipe (GDGM 45266) **b** Unchanged pileus surface and pale blue (in some patches) hymenophore when injured (GDGM 45266) **c** Pale bluing hymenophore (after injured) with reduced stipe (GDGM 45913, holotype) **d** Dark brownish hymenophore after exposed to air (GDGM 45913, holotype).

**Figure 5.**
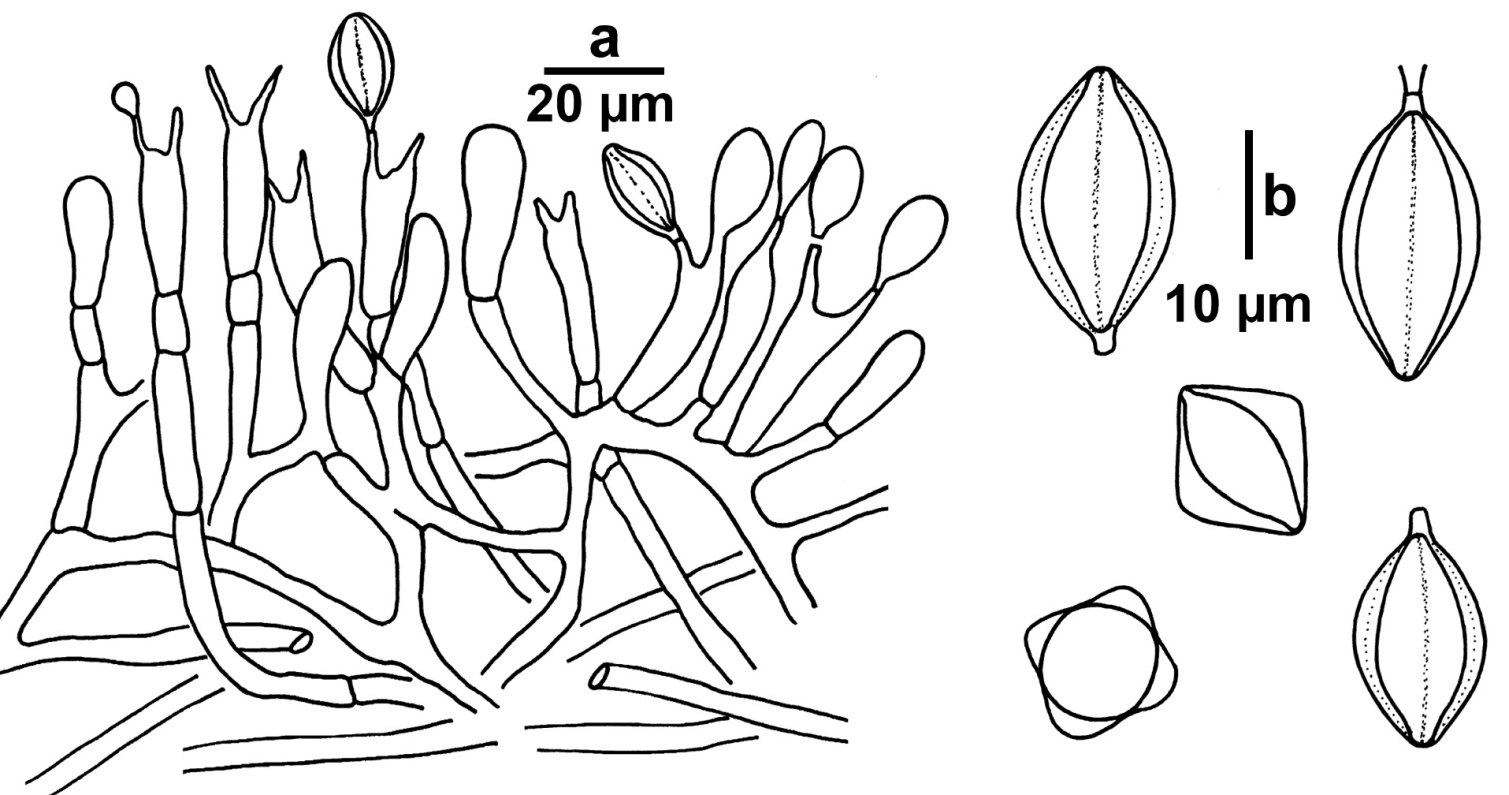
Microscopic features of *Rossbeeveragriseobrunnea*. **a** Basidia and basidioles development at different stages (GDGM 45266) **b** Basidiospores in different views (star-shaped in side view and quadrangular in polar view) (GDGM 45913, holotype).

**Figure 6.**
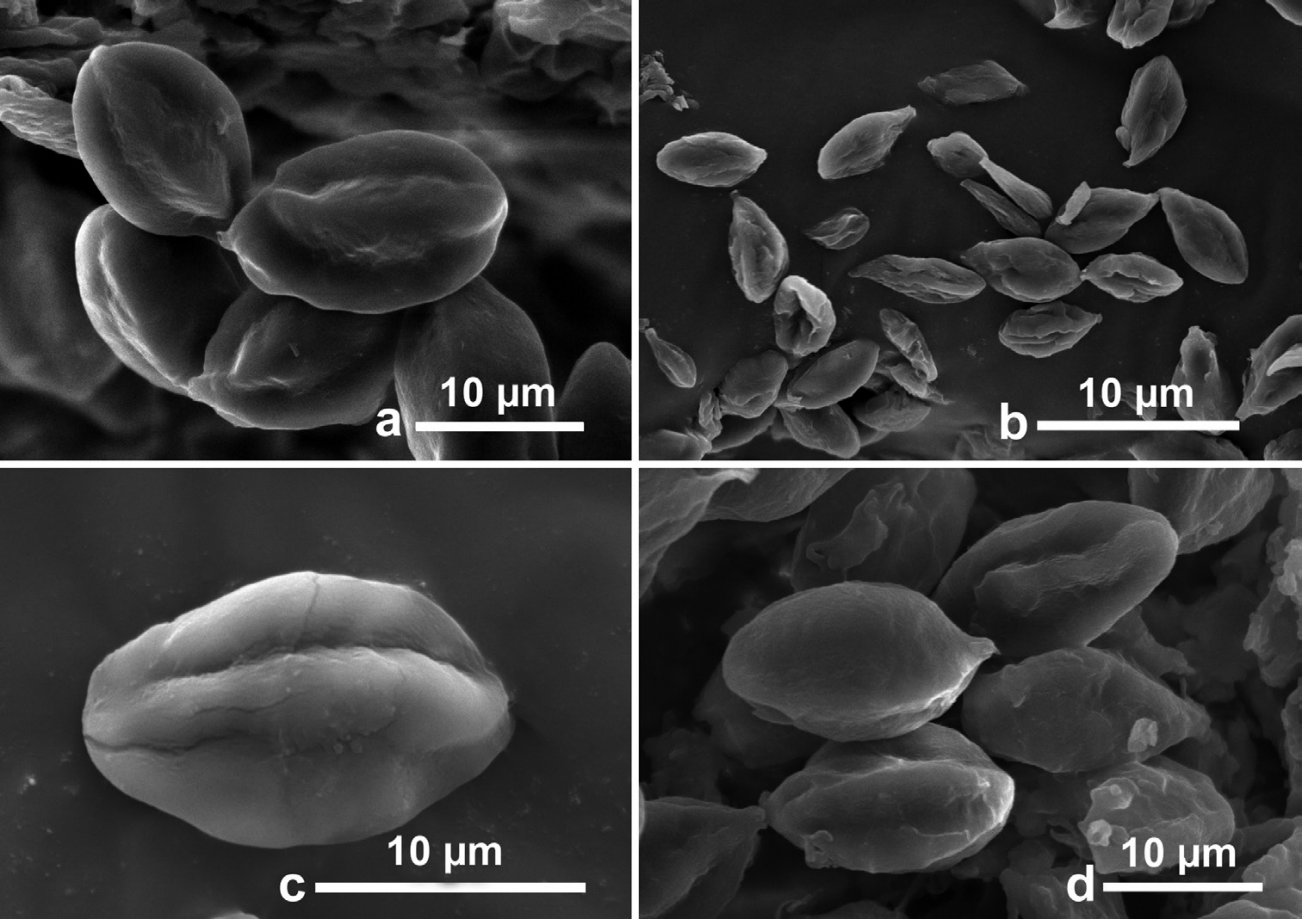
SEM of basidiospores of *Rossbeevera* species. **a, b***Rossbeeverabispora* (**a**GDGM 45612 **b**GDGM 45639) **c, d***Rossbeeveragriseobrunnea* (**c**GDGM 45266 **d**GDGM 45913, holotype).

## Discussion

Most of the species within *Rossbeevera* share common features like globose to subglobose sequestrate basidiomata with bluish discoloration (due to oxidation of pulvinic acid) when injured (either pileus or hymenophore), usually 1–2-spored but sometimes 4-spored basidia, ornamented basidiospores with 4–5 longitudinal ridges (star-shaped in cross section), absence of hymenial cystidia (except *R.griseovelutina*), and loose arrangement of hymenophoral trama with 2–5 μm wide hyphae. However, continental distance, habitat with different hosts, molecular data or genetic distance and some macro- and micro-morphological differences make them distinct species within *Rossbeevera*.

In the protologue, the basidiospore size of *R.bispora* is 15–21 × 10–12 μm (Zhang and Yu 1989). Our re-examination of the type material of *R.bispora* (GDGM 5688) showed that the basidiospore size is 16–21 × 10–12 μm [mean 18.35 × 11.02 μm, Q = [1.56–1.72(–1.81), Q_m_ = 1.66 ± 0.11], which is similar to that of the original description. The Q values [(1.63–1.83(–1.90), Q_m_ = 1.75 ± 0.11] of basidiospores derived from the new collections are slightly higher than the type material of *R.bispora*. However, the color changes of the pileus and hymenophore, 2-spored basidia and their association with broad-leaved trees suggest that the modern collections (including those from the type locality, Dinghushan Nature Reserve) are conspecific with *R.bispora*. Extraction of DNA sequences from the type material of *R.bispora* (GDGM 5688) was not successful due to the poor quality of the DNA from the aged specimen (collection date: October 13, 1982; Zhang and Yu 1989). For examination of evolutionary relationships within *Rossbeevera* and phylogenetic stability of this species we provide DNA sequences derived from the new collections. Prior to this study, *R.bispora* was known only from the type locality (Guangdong Province: Dinghushan Biosphere Reserve Forest), but we demonstrate it has a wide geographic distribution in south China (Guangdong Province: Baiyun Mountain and Tianluhu Park).

The rusty brown to dark brown or chocolate brown hymenophore (after exposure to air for 3–5 minutes or at maturity) in *R.griseobrunnea* occurs also in *R.vittatispora*, *R.pachydermis* and *R.griseovelutina* Orihara. However, *R.vittatispora*, originally described from Australia has a white to pale grayish to buff pileus staining greenish blue or indigo blue in some patches on the surface and shorter and narrower basidiospores measuring 9–12(–13) × 4–5.5(−6) μm (Lebel et al. 2012). *Rossbeeverapachydermis*, originally described from New Zealand, has a restricted distribution to that country and differs from the new species in having large basidiomata (up to 50 mm broad), relatively smaller basidiospores measuring 11–14 × 8–10 µm, and is mainly associated with *Nothofagus* (Lebel et al. 2012). The East Asian *R.griseovelutina* is distinctive on account of its velvety basidiomata, abundant hymenial cystidia, trichodermal elements in the pileus, and relatively longer basidiospores 14.4–31.9 × 6.7–10.4 μm (Lebel et al. 2012, Orihara et al. 2012). Phylogenetically, *R.paracyanea*, originally described from Japan, is a close sister species to *R.griseobrunnea* with moderate support value (68% MLBS, Fig. 1), but significantly differs from the latter species in having white to grayish basidiomata when young, becoming blue-gray to dark gray with age, an off-white hymenophore when young that turns indigo blue very quickly and strongly when touched or exposed to air, relatively narrower basidiospores (14–19.3 × 6.0–9.2 μm), and it occurs with *Quercusgilva* Blume and *Castanopsiscuspidata* Schottky (Orihara et al. 2016).

Besides the comparisons with the closely related species of *Rossbeevera*, two known Chinese species, *R.bispora* and *R.yunnanensis* can be compared with *R.griseobrunnea*. Both *R.griseobrunnea* and *R.bispora* share 2-spored basidia, brown to dark brown hymenophore at maturity, and are putatively associated with broad-leaved trees. However, *R.bispora* can be differentiated from *R.griseobrunnea* by the deep bluing reaction of the pileus and hymenophore when bruised or exposed to air (Zhang and Yu 1989) and it is also a phylogenetically distinct species (Fig. 1). *Rossbeeverayunnanensis*, known as the earliest divergence lineage within *Rossbeevera*, is distinguished from *R.griseobrunnea* in having a very thin, whitish pileus which becomes blue-green when injured and a reddish brown to blackish brown hymenophore at maturity (Orihara et al. 2012, Orihara 2018). Apart from China, *R.yunnanensis* is known also from Japan which is about 3150 km from the type locality (Chuxiong, Yunnan Province, China vs Hiroshima Prefecture, Japan) (Orihara 2018), suggesting that the species has a wide geographic distribution.

### Key to the taxa *Rossbeevera* known from Northern Hemisphere (China, Japan and Singapore/Malaysia) and Southern Hemisphere (Australia and New Zealand)

**Table:** 

1	Geographical distribution- Southern Hemisphere (Australasia)	**2**
–	Geographical distribution- Northern Hemisphere (Asia)	**4**
2	Distributed in Australia, basidiospores within 9–15 × 3–6 μm	**3**
–	Distributed in New Zealand, basidiospores 11–14 × 8–10 μm, associated with mainly *Nothofagus* spp., no grayish tints on the surface	*** R. pachydermis ***
3	Basidiospores 12–14 × 3–4.5 μm, basidiomata turn deep blue on bruising, restricted to western Australia	*** R. westraliensis ***
–	Basidiospores 9–12 × 4–5.5 μm, basidiomata turn blue to deep blue in some patches, widespread in eastern Australia	*** R. vittatispora ***
4	Distributed in East Asia	**5**
–	Distributed in Southeast Asia (Singapore/Malaysia), (13–)15–17 × (7–)8–9 μm, with Q values = 1.76–2.05	*** R. mucosa ***
5	Distributed in both Japan and China	*** R. yunnanensis ***
–	Distributed either in Japan or China	**6**
6	Basidia constantly 1 or 2-spored, either dark/strong or partial bluing pileus and hymenophore	**7**
–	Basidia 2-, 3-and 4-spored, bluing pileus and hymenophore	**8**
7	Pileus and hymenophore not bluing or partially pale bluing when injured or exposed to air, turn rusty brown to dark brown after exposure to air for a long time, found in China	*** R. griseobrunnea ***
–	Pileus and hymenophore bluing, basidiospores 15–21 × 10–12 μm, found in China	*** R. bispora ***
8	Strong bluing reaction, basidiospores mean 5–18 × 6.5–9.4 μm	**9**
–	Strong bluing reaction, velvety pileus, cystidia present, basidiospores mean >22.2 × 8.7 μm, found in Japan	*** R. griseovelutina ***
9	Hilar appendages (HA) 1.6–3.2 μm, basidiospores Q_m_ = 1.9, found in Japan	*** R. eucyanea ***
–	Hilar appendages 2.1–5.9 μm, basidiospores Q_m_ = 2.1–2.4	**10**
10	Hilar appendages 2.1–4.5 μm, basidiospores 14–19.3 × 6.9–9.2 μm (mean 16.7 × 8 μm), found in Japan	*** R. paracyanea ***
–	Hilar appendages 2.4–5.9 μm, basidiospores 13.4–18.3 × 5.8–7.3 μm (mean 15.8 × 6.5), found in Japan	*** R. cryptocyanea ***

## Supplementary Material

XML Treatment for Rossbeevera bispora

XML Treatment for Rossbeevera griseobrunnea
